# Retraction Note: The application of non-uniform magnetic field for thermal enhancement of the nanofluid flow inside the U-turn pipe at solar collectors

**DOI:** 10.1038/s41598-024-79444-6

**Published:** 2024-11-19

**Authors:** Sida Li, Liudan Mao, As’ad Alizadeh, Xin Zhang, S. Valiallah Mousavi

**Affiliations:** 1 Key Laboratory of Crop Harvesting Equipment Technology of Zhejiang Province, Jinhua Polytechnic, Jinhua, China; 2 Chongqing Water Conservancy & Electric Power Construction Survey & Design Research Institute Hangzhou Branch, Hangzhou, China; 3 Department of Civil Engineering, College of Engineering, Cihan University-Erbil, Erbil, Iraq; 4 Zhejiang Tongjing Technology Co. Ltd, Quzhou, China; 5 Department of Mechanical Engineering, Technical and Vocational University (TVU), Tehran, Iran

Retraction of: *Scientific Reports* 10.1038/s41598-023-35659-7, published on 25 May 2023.

The Editors have retracted this Article because an investigation by the Publisher found irregularities with respect to submission and authorship.

Sida Li, Liudan Mao and Xin Zhang disagree with this retraction. As’ad Alizadeh did not respond to correspondence from the Editor about this retraction. S. Valiallah Mousavi did not state explicitly whether he agrees to this retraction.

